# PIGNON: a protein–protein interaction-guided functional enrichment analysis for quantitative proteomics

**DOI:** 10.1186/s12859-021-04042-6

**Published:** 2021-06-04

**Authors:** Rachel Nadeau, Anastasiia Byvsheva, Mathieu Lavallée-Adam

**Affiliations:** Department of Biochemistry, Microbiology and Immunology, and Ottawa Institute of Systems Biology, Faculty of Medicine, University of Ottawa, 451 Smyth Road, Room 4170, Ottawa, ON K1H 8M5 Canada

**Keywords:** Protein–protein interactions, Graph theory, Quantitative proteomics, Functional enrichment analysis, Network biology, Differential expression, Breast cancer

## Abstract

**Background:**

Quantitative proteomics studies are often used to detect proteins that are differentially expressed across different experimental conditions. Functional enrichment analyses are then typically used to detect annotations, such as biological processes that are significantly enriched among such differentially expressed proteins to provide insights into the molecular impacts of the studied conditions. While common, this analytical pipeline often heavily relies on arbitrary thresholds of significance. However, a functional annotation may be dysregulated in a given experimental condition, while none, or very few of its proteins may be individually considered to be significantly differentially expressed. Such an annotation would therefore be missed by standard approaches.

**Results:**

Herein, we propose a novel graph theory-based method, PIGNON, for the detection of differentially expressed functional annotations in different conditions. PIGNON does not assess the statistical significance of the differential expression of individual proteins, but rather maps protein differential expression levels onto a protein–protein interaction network and measures the clustering of proteins from a given functional annotation within the network. This process allows the detection of functional annotations for which the proteins are differentially expressed and grouped in the network. A Monte-Carlo sampling approach is used to assess the clustering significance of proteins in an expression-weighted network. When applied to a quantitative proteomics analysis of different molecular subtypes of breast cancer, PIGNON detects Gene Ontology terms that are both significantly clustered in a protein–protein interaction network and differentially expressed across different breast cancer subtypes. PIGNON identified functional annotations that are dysregulated and clustered within the network between the HER2+, triple negative and hormone receptor positive subtypes. We show that PIGNON’s results are complementary to those of state-of-the-art functional enrichment analyses and that it highlights functional annotations missed by standard approaches. Furthermore, PIGNON detects functional annotations that have been previously associated with specific breast cancer subtypes.

**Conclusion:**

PIGNON provides an alternative to functional enrichment analyses and a more comprehensive characterization of quantitative datasets. Hence, it contributes to yielding a better understanding of dysregulated functions and processes in biological samples under different experimental conditions.

**Supplementary Information:**

The online version contains supplementary material available at 10.1186/s12859-021-04042-6.

## Background

High throughput quantitative proteomics studies provide an overview of the activity of different processes and functions in a biological sample. Mass spectrometry-based quantitative proteomics approaches are routinely used to quantify proteins in biological samples [1–4]. Strategies involving both stable isotope labelling [5–8] and label-free mass spectrometry [9] can be used to compare the expression of proteins across different samples and experimental conditions. When contrasting protein expression in different experimental conditions, it is common to use univariate statistical tests, such as the Student’s *t *test and Mann Whitney U test to assess the significance of protein differential expression. Arbitrary thresholds are then typically established (e.g. a *p *value < 0.05, and/or expression fold-change ≥ 1.5 or ≤ 0.$\overline{6}$) to determine the set of proteins deemed differentially expressed. Functional enrichment analyses are then often applied to the set of differentially expressed proteins in order to provide insights into the role they play in the biological samples and about the molecular impacts of the studied experimental conditions. Functional annotations investigated may take the shape of Gene Ontology (GO) terms [10], KEGG [11] and REACTOME [12] pathways, or MSigDB signatures [13].

Computational tools such as Ontologizer [14], GoMiner [15], and DAVID [16] are often applied to compare the occurrences of functional annotations among the set of significantly differentially expressed proteins against all of those in the entire set of quantified proteins using statistical tests such as the Fisher’s exact test to determine the significance level of the enrichment. Such a functional enrichment strategy is limited by the use of arbitrary cut-offs determining whether a protein is significantly differentially expressed or not. Two proteins may achieve very similar significance values, but if they sit on different sides of the established cut-offs, they end up being treated very differently, with one being considered for functional enrichment and the other not. Furthermore, many proteins sharing a given annotation may show some level of differential expression, but not sufficient to pass the chosen threshold. Such an annotation would therefore not be considered by the functional enrichment analysis even though a moderate differential expression of many proteins in a given pathway could contribute to the dysregulated phenotype. Methods such as Gene Set Enrichment Analysis (GSEA) [13] and GOrilla [17] have been proposed to counter such a loss of information by assessing whether genes sharing a given annotation are enriched towards the top or bottom of a ranked list of genes based on a given property of interest, such as differential expression. Similarly, PSEA-Quant [18, 19] was also proposed to evaluate the enrichment of a given annotation among abundant proteins that are reproducibly quantified in a set of replicate mass spectrometry analyses. Such methods are capable of detecting annotations for which the members are showing a moderate level of differential expression, but not necessarily a significant one for most individual genes or proteins. Nevertheless, some of the annotations can be quite general. Indeed, GO include terms such as “cytoplasm” or “metabolism”. The relationship between the proteins that share such annotations can be quite weak. One might therefore be more interested in proteins that do not only share a given annotation and show some degree of differential expression, but also have some sort of relationship, such as interacting together or being part of a given molecular pathway or protein complex.

Relationships between proteins can be derived from protein–protein interactions (PPIs). Indeed, proteins typically interact with one another to perform their functions, whether those interactions are transient or form protein complexes. Methods such as yeast two-hybrid [20], affinity purification [21, 22] and proximity labelling coupled to mass spectrometry [23], and have led to the mapping of large networks of PPIs [24–28]. Such PPI networks can therefore help to guide functional enrichment analyses by providing a structure to the data and focusing their results on annotations involving proteins with some level of relationships. Interesting relationships could be represented by proteins that are densely connected or clustered in the PPI network. We have previously developed an approach named GoNet that detected GO terms that annotate proteins that are significantly clustered in PPI networks [29]. Furthermore, PPI networks are not the only types of biological networks that can provide additional information in the context of functional enrichment analyses. Alexeyenko et al. proposed a Network Enrichment Analysis (NEA), which investigates the connectivity in biological networks of genes or proteins that are significantly differentially expressed in a quantitative dataset under different experimental conditions [30, 31]. Such groups of genes or proteins are of great interest since they share a relationship, such as PPIs or a cellular localization, depending on the type of biological network used as input. However, this approach still suffers from the same limitations as standard functional enrichment analyses, since it relies on arbitrary significance cut-offs to determine a set of differentially expressed genes or proteins and on an individual assessment of the significance level for each gene or protein. Similarly, EnrichNet evaluates a graph theory-based statistic for a set of genes or proteins of interest (e.g. significantly differentially expressed) in a molecular network and provides a visualization of sub-networks of interest that are associated with a given annotation involving the inputted genes or proteins [32]. Much like NEA, it also relies on a list of genes or proteins of interest that is defined using some arbitrary threshold and requires a minimum of 10 genes or proteins as input, thereby limiting the usage of the approach. Similarly, Signorelli et al. proposed NEAT, another network-based enrichment approach relying on a list of genes of interest determined using a cut-off [33]. Interestingly, NEAT however has a short running time and can be applied in networks in which edges are directed (i.e. the relationship between genes or proteins is directional, such as a gene regulating the expression of another), unlike most methods. Finally, more recently, NGSEA was proposed to address the limitation of a selection of a gene set of interest for which the enrichment analysis must be performed [34]. Much like GSEA, NGSEA performs a ranking of genes based on a property of interest. NGSEA ranks a given gene based on its differential expression ratio in different experimental conditions, but also integrates in this ranking criterium the differential expression ratios of its immediate neighbors in a molecular network. However, NGSEA makes a limited use of the analyzed molecular networks by only considering the direct interactors of a gene and does not take advantage of other genes that may be in the vicinity. Such other genes could be part of the same pathway without such a direct relationship. The sole integration of direct relationships also makes NGSEA less effective when using incomplete networks that comprise large numbers of false negative relationships.

Herein, we propose a novel alternative approach for functional enrichment analysis: PIGNON, a Protein–protein Interaction-Guided fuNctiOnal eNrichment analysis for quantitative proteomics datasets. PIGNON investigates whether the proteins annotated by a given GO term are significantly clustered within a PPI network that is weighted by the level of differential expression of its constituting proteins. PIGNON does not require the identification of a set of proteins that are differentially expressed according to some threshold and explores the entirety of the PPI network topology when assessing the enrichment of a given GO term. We applied this approach to a quantitative proteomics analysis of different breast cancer subtypes from Tyanova et al. [35] We compared the results of PIGNON against those of a standard functional enrichment analysis techniques, an approach performing functional enrichment analysis on protein clusters derived from the Markov Clustering algorithm [36], the Gene Set Enrichment Analysis (GSEA) algorithm [13], as well as the NEA biological network-based functional enrichment analysis [30, 31]. We show that our tool uniquely detects annotations whose associated proteins are significantly clustered and show a differential level of expression. Indeed, PIGNON identifies dysregulated functional annotations in the hormone receptor positive (HR+), human epidermal growth factor receptor 2 (HER2+) and triple negative (TN) breast cancer subtypes, providing insights into the molecular mechanisms of these conditions. These results demonstrate that PIGNON represents an alternative tool to rudimentary and sophisticated functional enrichment analyses and that it can provide a more comprehensive understanding of biological samples analyzed using quantitative proteomics.

## Results

**Fig. 1:**
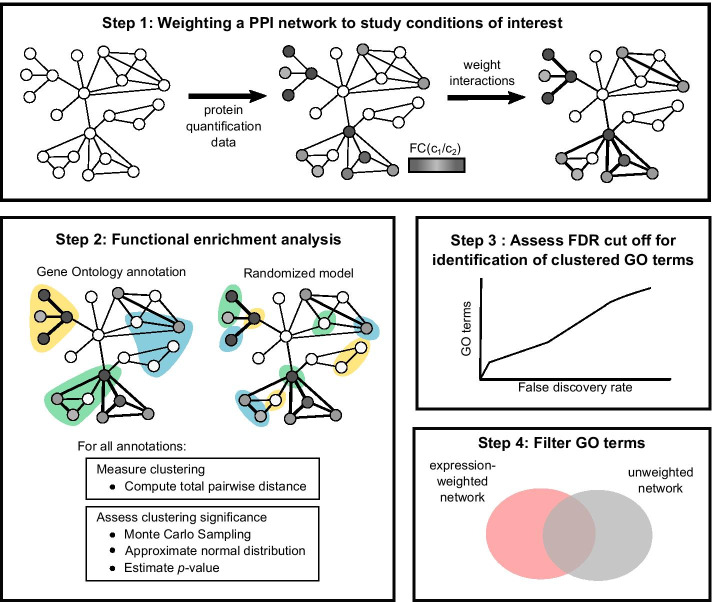
Illustrated overview of PIGNON. *Step 1* PIGNON builds a graph representation of the protein–protein interaction (PPI) network. Here, every node is a protein, and the edges between them a PPI. Edges can be weighted based on the fold-change (FC) of the protein expression measured between condition 1 (c_1_) and condition 2 (c_2_). *Step 2* PIGNON annotates the proteins of the PPI network using Gene Ontology terms, and in parallel, the network is annotated with a shuffled set of these annotations. Here, annotations are represented by the various colours (blue, yellow and green). The clustering of proteins associated with a given annotation is measured using the Total pairwise distance (TPD). A Monte Carlo sampling approach followed by an approximation using normal distributions is then performed to assess the clustering statistical significance. *Step 3* We assess the clustering confidence through a false discovery rate estimation. *Step 4* Significantly dysregulated GO terms in the expression-weighted PPI network are reported following filtering of significant GO terms identified in the unweighted PPI network

We present a novel functional enrichment analysis tool named PIGNON, which identifies functional annotations for which the proteins are differentially expressed and clustered in a PPI network. To demonstrate PIGNON’s capabilities, we analyzed a quantitative proteomics dataset from Tyanova et al. [35], where breast cancer tumours from the HER2+, HR+ and TN subtypes were analyzed. We integrated this dataset with two PPI networks derived from the BioGRID [37] and STRING [38] repositories. The BioGRID PPI network contained 266,136 PPIs involving 16,563 proteins, while the STRING PPI network included 959,614 PPIs implicating 19,028 proteins (see Methods for processing steps). Protein expression values are used to weight PPIs in these networks such that interacting partners with similar levels of differential expression become “closer” in the network. The clustering of 14,211 GO terms was investigated by PIGNON in the BioGRID PPI network and 14,273 GO terms in the STRING PPI network. Figure 1 provides a graphical representation of PIGNON’s methods.

### PIGNON’s normal approximation provides accurate clustering *p *values

Since the Monte Carlo sampling approach can hardly estimate the *p *values of very small clusterings with a *p* value < 10^–7^, PIGNON estimates a null model of clustering for each protein set sizes using a normal distribution. When comparing the distribution of total pairwise distances (TPD) obtained using Monte Carlo sampling to the one generated from a normal approximation, we see that the normal approximation can be a good estimate of the Monte Carlo-derived distribution in the BioGRID PPI network. While the fit between the two distributions is not ideal for a small protein set size such as 5, the normal distribution appears to be a tight upper bound on the probability of strong clusterings (i.e. small TPDs) (Additional file 1: Figure S1A and S1B). It therefore yields a conservative estimate of the clustering *p *values, which is desirable. The fit between the two distributions also appears to increase as the protein set size grows (Additional file 1: Figure S1C–F). Given these results, we chose to estimate clustering significance *p *values using the normal approximation of the TPD null distributions.

### PIGNON can accurately estimate the statistical significance of clusterings

**Fig. 2:**
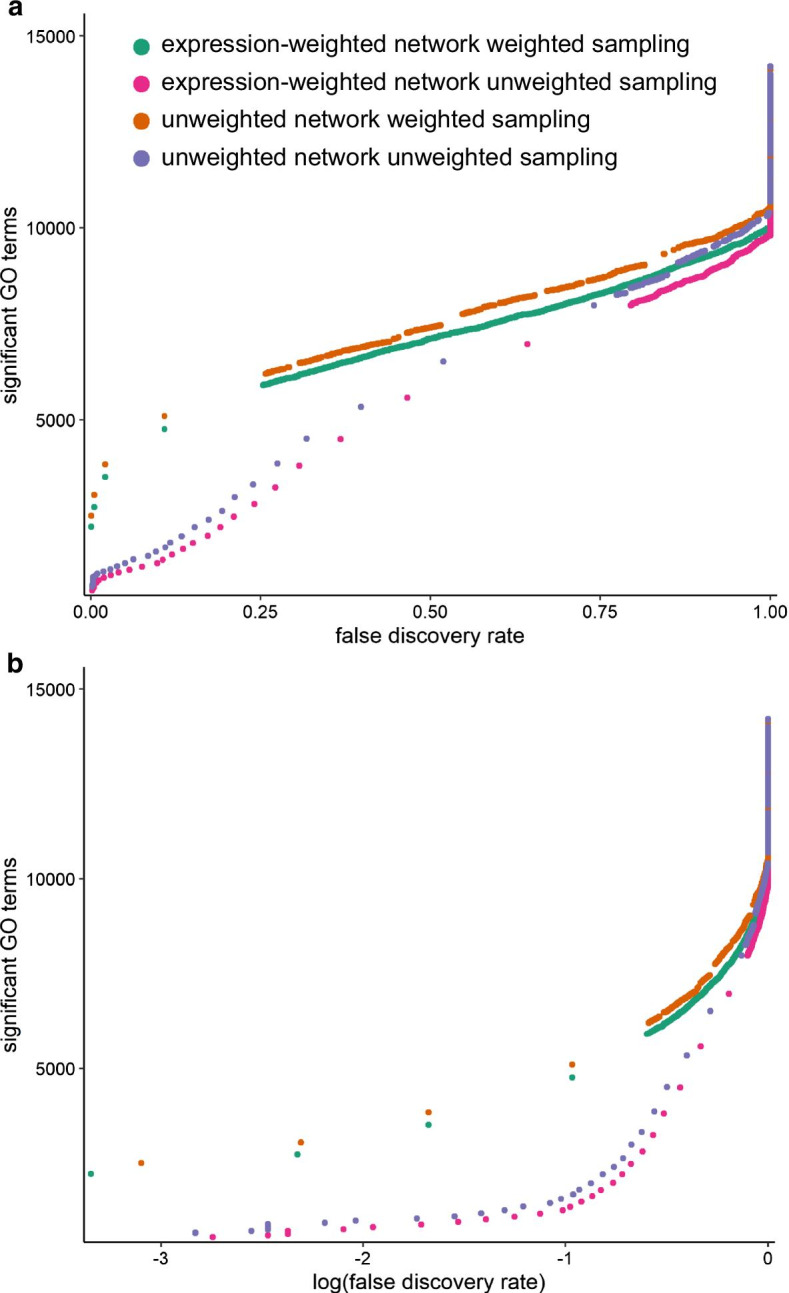
PIGNON identifies significant GO terms using different sampling approaches and different versions of the BioGRID PPI network. Number of significant GO terms identified at various **a** FDR and **b** logged (base 10) FDR cut-offs for the HER2+/TN expression-weighted and unweighted BioGRID networks analyzed with PIGNON using both the unweighted and weighted Monte Carlo sampling strategies

Given the fact that PIGNON’s *p *values are estimations from a normal approximation, we estimate the false discovery rate (FDR) associated with a given *p *value significance threshold (see Methods). We compared the FDRs associated with the *p *value significance threshold achieved by PIGNON using both sampling strategies (unweighted and weighted) and both types of BioGRID networks (expression-weighted and unweighted). As expected, the FDRs drop as *p *values decrease for all approaches. (Additional file 2: Figure S2) Here, the performances of PIGNON when processing the expression-weighted and unweighted networks are similar, which indicates that the weighting of PPIs does not greatly alter the accuracy of the *p *value estimations. Interestingly, at the same *p *value threshold, the FDRs of the weighted sampling approach are significantly lower than those of the unweighted sampling method. For instance, the weighted sampling approach yields a FDR of 0.004 at a *p *value threshold of 0.0014 when analyzing the expression-weighted network comparing the HER2+ and TN subtypes, while the unweighted sampling method yields a FDR of 0.37 at a similar *p *value cut-off of 0.0015. This result is likely indicating that the weighted-sampling approach is a more accurate null model for clustering significance estimation. We observe a similar behaviour when we compare the number of significantly clustered GO terms under a given FDR threshold (Fig. 2). Specifically, the number of significant GO terms at various FDR levels are comparable when PIGNON analyzes expression-weighted and unweighted BioGRID networks, while the weighted sampling approach identifies more significant GO terms than the unweighted sampling approach at similar FDR levels. For instance, the weighted sampling detected 2212 significantly clustered GO terms in the expression-weighted network of HER2+ subtype compared to TN at a FDR < 0.001 (Additional file 3: Table S1). At a similar FDR threshold (< 0.0018), the unweighted sampling approach only detected 517 clustered GO terms in the same network (Additional file 4: Table S2). These results indicate that PIGNON can reliably identify GO terms that are significantly clustered in the PPI network. A similar trend is observed when analyzing the unweighted BioGRID PPI network (Additional file 5: Table S3, Additional file 6: Table S4).

### PIGNON detects functional annotations for which the associated proteins are clustered and dysregulated in the PPI network

**Fig. 3:**
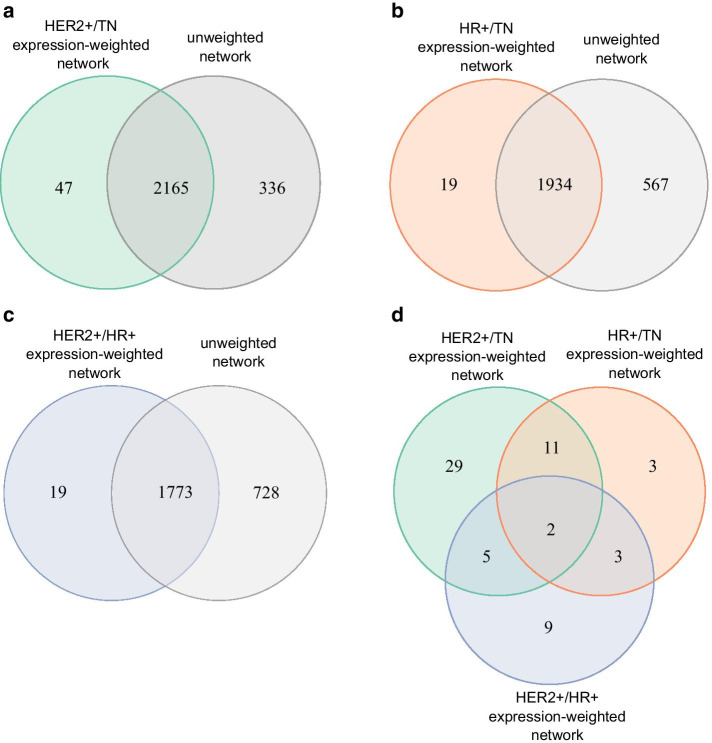
Filtering significant GO terms identified by PIGNON enables the identification of dysregulated biological processes in breast cancer subtypes. Venn diagram comparing the significant GO terms identified in the unweighted BioGRID network to **a** the HER2+/TN (FDR < 0.001), **b** the HER2+/HR+ (FDR < 0.0029), and **c** the HR+/TN (FDR < 0.0013) expression-weighted BioGRID networks using weighted Monte Carlo sampling. **d** Venn diagram comparing the overlap between GO terms only found to be significant in the expression-weighted BioGRID networks comparing the different breast cancer subtypes

Since PIGNON evaluates for a functional annotation both the clustering of its associated proteins and their level of dysregulation, we looked to assess the role of the PPI network connectivity on the identification of a significant GO term. This is important, because proteins that are annotated with the same GO term are likely to be more clustered than a randomly selected set of proteins, since they are likely involved in similar biological processes, part of the same protein complex or co-localizing in a given cell compartment. To do so, we compared the GO terms identified with our approach using the expression-weighted and unweighted BioGRID PPI networks at similar significance threshold. For all three breast cancer subtype comparisons, the majority of the GO terms detected were also found to be clustered in the unweighted network. Specifically, at a FDR < 0.001, 2212 GO terms were detected in the HER2+/TN expression-weighted PPI network, while 2165 of these GO terms were detected to be clustered by PIGNON in the unweighted network at the same FDR threshold (Fig. 3a). Similar results are obtained when comparing the other breast cancer expression-weighted networks to their unweighted counterparts. At a FDR < 0.0029, 1934 GO terms were found to be clustered in the unweighted network out of the 1953 GO terms detected in the HR+/TN expression-weighted network. (Fig. 3b) (Additional file 7: Table S5). On the other hand, 1773 of the 1792 GO terms detected in the HER2+/HR+ expression-weighted network were also clustered in the unweighted network at a FDR < 0.0013 (Fig. 3c) (Additional file 8: Table S6). These shared GO terms are likely consequences of the innate clustering of their annotating proteins in the PPI network and display a bias in GO term clustering in the network. Hence, the 47 GO terms that are unique to PIGNON’s analysis of the HER2+/TN expression-weighted network are likely consequent of the dysregulation of the annotated proteins in these breast cancer subtypes. The same applies to the two sets of 19 GO terms that are unique in the PIGNON analysis of the HR+/TN and HER2+/HR+ expression-weighted PPI networks. Of note, the above FDR thresholds for the different breast cancer subtype comparison consist of the lowest FDR above zero that could be estimated by PIGNON in the respective expression-weighted networks (i.e. the highest level of confidence).

### PIGNON identifies functional annotations using the STRING PPI network.

To assess PIGNON’s generalizability, we ran its analysis on the STRING PPI network. In this network, 7225, 5935 and 7391 GO terms were found to be significantly clustered (FDR < 0.001), respectively in the HER2+/TN, HER2+/HR+, and HR+/TN+ expression-weighted STRING PPI networks (Additional Files 9, 10, 11, Tables S7–9). When filtered against the 8296 significantly clustered GO terms identified at a FDR < 0.001 in the unweighted STRING PPI network (Additional file 12: Table S10), 138, 29 and 160 GO terms were unique to the PIGNON’s analysis of the HER2+/TN, HER2+/HR+ and HR+/TN expression-weighted STRING PPI networks, respectively (Additional Files 13, 14, 15, Figures S3–5). These results show that PIGNON is equally able to identify significantly dysregulated pathways in the BioGRID and STRING expression-weighted networks.

Furthermore, we compared the GO terms identified uniquely in the expression-weighted network of both BioGRID and STRING. In the HER2+/TN networks, “ErbB-3 class receptor binding”, was highlighted from both networks. In addition, the HR+/TN expression-weighted BioGRID and STRING networks both guided the discovery of the “cell–cell junction” cellular component annotation. Interestingly, both of these GO terms have been well characterized to play a role in breast cancer [39, 40]. These results also suggest that as expected, the annotations identified by PIGNON may change based on the type of PPI network used and its topology.

### PIGNON detects functional annotations that are not highlighted by standard approaches.

We first benchmarked our approach against a standard statistical pipeline, where the significance of differential protein expression is assessed using a Student’s *t* test, and a GO enrichment analysis is then performed using Ontologizer [14] on the significantly differentially expressed proteins (see Methods). This approach deemed 3 proteins to be significantly differentially expressed between HER2+ and TN as well as 4 and 0 between HER2+/HR+ and HR+/TN, respectively, at a FDR-adjusted *p *value < 0.05 (Additional file 16: File S1). From these proteins, no GO terms were found to be enriched among the differentially expressed proteins between any of the conditions (Additional file 17: File S2). In order to enable a comparison with PIGNON’s results, we loosened up the cut-off for protein differential expression to a FDR-adjusted *p *value ≤ 0.2 and a fold-change ≤ 0.$\overline{6}$ or ≥ 1.5. With these criteria 13, 19 and 34 proteins were deemed differentially expressed between the HER2+/TN, HER2+/HR+, and HR+/TN conditions, respectively (Additional file 16: File S1). No GO terms were found to be significantly enriched among the proteins differentially expressed when comparing the HER2+/TN or the HER2+/HR+ conditions using the Ontologizer method. Three GO terms, “regulation of protein binding”, “regulation of binding”, “catalytic activity, acting on DNA” were highlighted in the comparison of the HR+/TN subtypes at a FDR-adjusted *p* value < 0.05 (Additional File 18: File S3).

We also executed the GSEA algorithm [13] on the Tyanova et al. quantitative proteomics dataset. However, this approach did not reveal any significant GO terms in any of the three breast cancer subtype comparisons. (Additional file 19: File S4).

**Fig. 4:**
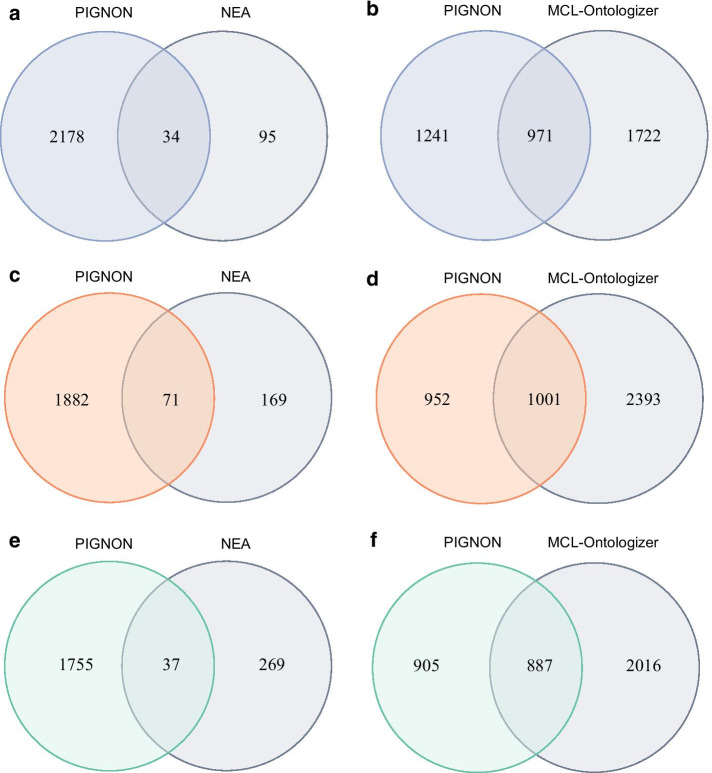
PIGNON identifies different GO terms than the NEA and MCL-Ontologizer approaches. Venn diagrams comparing the overlap of significant GO terms identified by PIGNON against the MCL-Ontologizer or NEA approach comparing the **a**, **b** HER2+/TN (PIGNON FDR < 0.001; MCL-Ontologizer FDR adjusted-*p* < 0.001; NEA FDR < 0.001), **c**, **d** HR+/TN (PIGNON FDR < 0.0029; MCL-Ontologizer FDR adjusted-*p* < 0.0029; NEA FDR < 0.0029), and **e**, **f** HER2+/HR+ (PIGNON FDR < 0.0013; MCL-Ontologizer FDR adjusted-*p* < 0.0013; NEA FDR < 0.0013) subtypes at comparable confidence thresholds

We then benchmarked PIGNON against the NEA [30, 31] network-based functional enrichment approach using the BioGRID PPI network. This analysis was performed on the same loose differential expression criteria that were used for the Ontologizer functional enrichment analysis (FDR-adjusted *p *value ≤ 0.20 and a fold change ≤ 0.$\overline{6}$ or ≥ 1.5) in order to supply sufficient input to the NEA algorithm. In this study, 129 GO terms were identified by NEA in the HER2+/TN conditions at an FDR < 0.001, 34 of which were also identified by PIGNON at the same threshold in the HER2+/TN expression-weighted BioGRID network (Fig. 4a). Similar results in terms of overlapping were obtained when contrasting the other conditions. Indeed, for the HR+/TN conditions 240 GO terms were identified by NEA (FDR < 0.0029) and 71 of those were also identified by PIGNON at the same threshold (Fig. 4c). Finally, for the HER2+/HR+ conditions, 300 GO terms were found to be significant by NEA (FDR < 0.0013), 37 of which were also identified by PIGNON at the same FDR (Fig. 4e) (for all results see Additional file 20: File S5). These results highlight that while both approaches shared a certain subset of GO terms that they deemed interesting, they still show a strong level of orthogonality in the reported results. This is likely due to the fact that NEA only considers a subset of the proteins from the quantitative proteomics studies in its analysis, while PIGNON uses the entire dataset.

Finally, we also benchmarked PIGNON against an in-house approach combining the Markov Clustering (MCL) algorithm [36] to identify protein clusters in the expression-weighted BioGRID PPI network and Ontologizer to detect GO enrichments among these clusters. This approach should therefore highlight GO terms that are clustered in such expression-weighted network. At comparable thresholds (i.e. FDR-adjusted *p* value < 0.001 and FDR < 0.001), the MCL-Ontologizer approach identified 2693 GO terms in the HER2+/TN expression-weighted network, while our approach identified 2212 GO terms, of which 971 of these GO terms were identified in both approaches (Fig. 4b) (Additional file 21: File S6). Hence, PIGNON detected 1067 GO terms deemed to be clustered in the HER2+/TN expression weighted network that this approach failed to detect. Similar results were obtained in the HR+/TN and HER2+/HR+ expression-weighted BioGRID networks. Specifically, 3394 GO terms were identified in the MCL-Ontologizer approach while 1953 GO terms were identified by PIGNON in the HR+/TN expression-weighted BioGRID network at comparable thresholds (i.e. FDR-adjusted *p* value < 0.0029 and FDR < 0.0029). In the HER2+/HR+ expression-weighted network, the MCL-Ontologizer approach identified 2903 GO terms, while PIGNON detected 1792 GO terms at comparable thresholds (i.e. FDR-adjusted *p* value < 0.0013 and FDR < 0.0013). This translated to 1001 and 887 GO terms identified by both approaches in the HR+/TN and HER2+/HR+ networks, respectively (Fig. 4d, f) (Additional file 22: File S7; Additional file 23: File S8). Interestingly, between the GO terms reported in both approaches 10, 4 and 7 GO terms were uniquely detected respectively in the HER2+/TN, HR+/TN and HER2+/HR+ expression-weighted networks, respectively compared to the unweighted network (Additional Files 24, 25, Figure S6–7). Of note, both approaches highlighted the “ADP metabolic process” GO term when comparing the HR+ and TN conditions, which was not clustered in the unweighted BioGRID network. The detection of such a GO term with both approaches is likely significant to the biology of the different conditions and has been implicated in breast cancer [41]. Overall, these results suggest that PIGNON highlights unique information about the dataset investigated that standard and currently used approaches do not. It further illustrates that it can serve as an additional approach in the data mining of quantitative datasets.

### PIGNON identifies GO terms that are known to be affected in different breast cancer subtypes

**Fig. 5:**
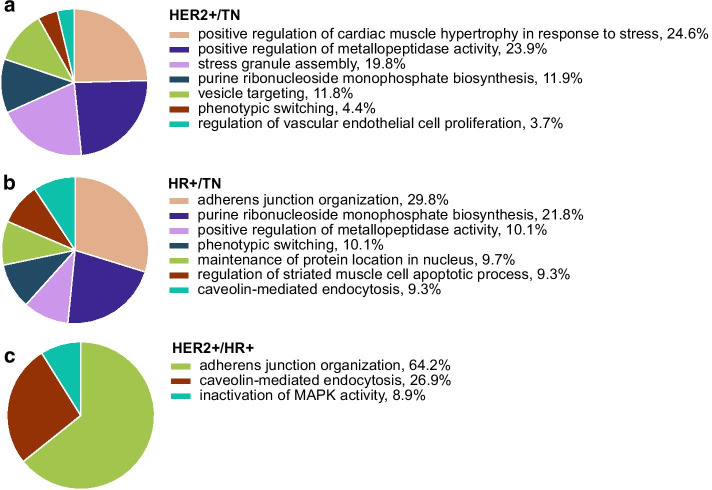
Biological processes identified by PIGNON in breast cancer subtype comparisons that were unique to the expression-weighted BioGRID networks. CirGO visualization of uniquely identified GO biological processes in **a** HER2+/TN (FDR < 0.001), **b** HR+/TN (FDR < 0.0029), **c** HER2/HR+ (FDR < 0.0013) expression-weighted BioGRID networks. The size of the pieces of the pies are proportional to the level of enrichment statistical significance and are also denoted as percentages next to the GO term names

To limit GO terms to those of the highest biological relevance to the studied breast cancer subtypes, we filtered the GO terms identified by PIGNON to those that are unique to the analysis of the expression-weighted networks when compared to that of the unweighted network. We identified 47 dysregulated GO terms between the HER2+ and TN subtypes (FDR < 0.001), 19 between the HER2+ and HR+ subtypes (FDR < 0.0013), and 19 as well between the HR+ and TN subtypes (FDR < 0.0029) (Fig. 3a–c). For the most part, the GO terms detected by PIGNON as being dysregulated have been previously characterized in breast cancers in the past, but have not necessarily been associated with these particular subtypes. Biological processes revealed in the HER2+ and TN subtypes included “vascular endothelial cell proliferation” and “stress granule assembly” (Fig. 5), while the HER2+ and HR+ subtypes showed a dysregulation of “Titin binding”, all of which are involved in tumorigenesis of breast cancers [42–44]. On the other hand, the comparison of HR+ and TN detected the “laminin-10 complex” term, for which downregulation is known in HR+ breast cancer [45]. (For cellular components, see Additional file 26: Figure S8; for molecular functions see Additional file 27: Figure S9).

We combined the results of the PIGNON analyses comparing the different breast cancer subtypes to compare the GO terms overlapping between subtypes and those that are unique to each breast cancer subtype (Fig. 3d). Common functional annotations to all breast cancer subtypes include “cell–cell junction” and “plasma membrane raft”, which are known factors of cancer progression [40]. Furthermore, PIGNON highlighted GO terms that were previously characterized in specific breast cancer subtypes, such as “epidermal growth factor receptor binding”, the “inactivation of MAPK activity” and “vesicle targeting” when analysing expression-weighted networks involving the HER2+ subtype [39, 46, 47]. PIGNON’s analysis of TN involved expression-weighted networks was characterized by “purine ribonucleotide triphosphate biosynthesis”, “regulation of metallopeptidase activity” and “cortical actin cytoskeleton” (Fig. 5), which have been shown to be major regulators of triple negative breast cancer pathogenesis [48–50]. Finally, the HR+ subtype was characterized by “adherence junction organization”, for which the dysregulation is implicated in lobular type breast cancers [51] and “caveolin-mediated endocytosis”, which is prevalent in non-HER2+ cancers [52]. Furthermore, our analyses revealed functional annotations related to vesicle transport and import, as well as stress responses that are known regulators of breast cancer progression [43, 47], but were not revealed by the original study conducted by Tyanova et al. It is worth noting that given the stringent FDR control imposed on PIGNON’s results (FDR < 0.001 for HER2+/TN, 0.0029 for HR+/TN, and 0.0013 for HER2+/HR +) one would expect roughly only 2, 6, and 2 of the GO terms identified by PIGNON’s analyses of each of the subtype comparisons, respectively to be false discoveries, since PIGNON reported 2212, 1953, 1792 in each comparisons. Hence, previously unreported functional associations with breast cancer subtypes reported by PIGNON are of high confidence.

## Discussion

### Alternative PPI weighting strategies

PIGNON weights PPIs in networks based on the fold-change of their interacting proteins between two compared experimental conditions. The algorithm treats both low and high fold-changes the same way. This approach enables us to look at both up- and down-regulation without prioritizing a given state and focuses on the detection of functional annotations that are simply dysregulated. An alternative method could have looked at these states independently. Indeed, PIGNON could be used to detect only annotations that are down-regulated and clustered in the network or, on the other hand, only up-regulated. Furthermore, the fold-change is not the only measure that could be used to integrate a notion of differential expression into a PPI network. For instance, the *t-*statistic or a *p *value resulting from a statistical test such as the Student’s *t* test could also be used as weights in a PPI network. Such measures have the advantage of capturing the variance in expression differences between the two samples.

### Alternative clustering measures

Alternative methods to the total pairwise shortest paths could have been used to provide a measure of clustering in a PPI network for the set of proteins annotated by a GO term. For instance, the sum of the weights of the minimum spanning tree of the proteins annotated by a GO term could be used instead. This would only consider a subset of the edges considered by the total pairwise shortest paths and would therefore be less sensitive to outliers that are very distant from the other proteins in the network. Nevertheless, the running time of this approach could become prohibitive, since minimum spanning trees need to be computed for each set of proteins being sampled, while the shortest path between all proteins is only computed once for a given network.

Both the shortest path-based measure and the minimum spanning tree approaches consider that all proteins contribute to the differential expression and clustering of a given annotation. However, not all proteins of a given annotation need to be dysregulated for the overall annotation to be significantly dysregulated. Therefore, a clustering measure that looked only at a fraction of the most clustered proteins in an annotation, similar to the approach adopted by LESMoN [53], may allow the detection of supplemental dysregulated annotations.

### Running time considerations and possible improvements.

Given the large number of samplings performed by PIGNON, its running time can be considerable. Here, we report the results of a Monte Carlo procedure sampling 10^7^ random protein sets for all protein set sizes. Reducing the number of samplings by 10- or even 100-fold can decrease the running time of the algorithm, while still enabling the approximation of normal distribution parameters. This parameter is adjustable in PIGNON’s implementation. Furthermore, in this analysis, we investigated GO terms that annotate as much as 1000 proteins. GO terms with such a large number of annotations are typically not of great biological interest. Reducing the maximum size of proteins sets for which PIGNON builds a null model using Monte Carlo sampling would therefore also decrease the running time of the algorithm. This is another parameter that is adjustable in PIGNON’s software package.

### PIGNON can be applied on any quantitative datasets

PIGNON was developed to perform functional enrichment analyses in quantitative proteomics datasets. We applied this method on a mass spectrometry investigation of breast cancer tumour subtypes. However, it can be used on any quantitative proteomics dataset that compares a least two conditions of interest. Furthermore, while the association between quantified proteins and PPI networks is natural, PIGNON could certainly be used to perform functional enrichment analyses in quantitative transcriptomics datasets, such as those generated using RNA-sequencing. Obviously, the expression of a transcript does not necessarily equate to the translation of its corresponding protein. Nevertheless, PIGNON could still be used to identify transcripts that are differentially expressed and for which the associated proteins are clustered in PPI networks. Such sets of transcripts would be likely to play an important role in the experimental conditions studied in the quantitative transcriptomics analysis. Finally, while this analysis focused on a human quantitative proteomics dataset, PIGNON can also analyze quantitative datasets originating from different species. The only requirement is that the PPI network used by PIGNON originates from the same species as the quantitative proteomics dataset.

## Conclusions

We have designed a novel PPI-guided functional enrichment analysis for quantitative proteomics datasets. Our approach, PIGNON represents an alternative strategy to identify significantly dysregulated functional annotations in between two different experimental conditions. Finally, PIGNON increases our ability to characterize quantitative proteomics datasets and therefore helps to provide a better understanding of the processes and mechanisms at work in biological samples under different conditions.

## Methods

### Method overview

Our approach, named PIGNON, identifies differentially expressed functional annotations by assessing the clustering of proteins associated with a given GO term within an expression-weighted PPI network. The network is weighted such that interacting proteins that are differentially expressed are connected using edges with a small weight. Our method measures the clustering of proteins sharing a given functional annotation by computing the total pairwise distance between the proteins. It then assesses the statistical significance of this clustering using a Monte Carlo sampling approach. Finally, it estimates a false discovery rate (FDR) for the annotations deemed significantly clustered in the expression-weighted network. Figure 1 provides a graphical representation of PIGNON’s methods.

### Quantitative proteomics dataset

To test our methods, we used a quantitative proteomics analysis of breast cancer tumours from three different subtypes performed by Tyanova et al. [35]. This study assessed the proteomics profile of whole tumours diagnosed as hormone receptor positive (HR+), human estrogen receptor 2 positive (HER2+), or triple negative breast cancer (TN) subtypes using super-SILAC coupled to mass spectrometry [54]. Weights are assigned to edges based on protein differential expression between two of the above breast cancer subtypes.

### Protein–protein interaction network and expression weighted interactions.

In the context of this study, we combined the above quantitative proteomics dataset with a human PPI network obtained either from the BioGRID PPI public repository (version 3.4.161) [37, 55], which contains 330,754 individual PPIs and 22,733 unique proteins, or from the STRING repository (version 11) [38], which encompasses 996,192 PPIs and 19,216 proteins. The following inclusion criteria were applied to generate the BioGRID and STRING PPI networks. Firstly, only human-to-human protein PPIs were considered, since the quantitative proteomics study involved human samples. Secondly, only PPIs that constituted the largest connected components of the networks were kept, a requirement of our statistical approach. Thirdly, only proteins that interacted with less than 1000 other proteins are kept in the networks, eliminating 12 and 189 overly connected proteins that condense and could mask clusters that are present the BioGRID and STRING networks respectively. In addition, to build the STRING network, only interactions with a medium confidence score above 0.4 were used. These filters resulted in a reduced network of 16,563 proteins involved in 266,136 PPIs for the BioGRID PPI network and 19,028 proteins involved in 959,614 PPIs for the STRING PPI network. These networks are represented as undirected graphs, $G = \left( {V, E} \right)$, where $v\in V$, the set of vertices (proteins) and $e\in E$, the set of edges connecting two vertices, (PPIs) ($e = v1,v2,$ where $v_{1} , v_{2} \in V$).

Based on the Tyanova et al. quantitative proteomics analysis, we assign weights to the edges of the networks as follows. The level of protein differential expression of a protein *v*_*i*_ is defined as the fold-change, $f\left( {v_{i} } \right)$. $f\left( {v_{i} } \right)$ is calculated as the ratio of the average expression of $v_{i}$ in one experimental condition (e.g. HER2+ tumours) over the average expression of $v_{i}$ in another condition (e.g. TN tumours) as provided by Tyanova et al.’s super-SILAC experiments. In the event that a protein in the network $v_{i}$ was not quantified by Tyanova et al., $f\left( {v_{i} } \right)$ was set to 1, indicating no differential expression. When comparing the HER2+/TN conditions 2883 proteins in the BioGRID PPI network have a $f\left( {{\text{v}}_{i} } \right)\neq 1,$ while 2597 and 2613 proteins were weighted in the HR+/TN and HER2+/HR+ networks respectively. For the STRING PPI network, 3897, 3740, 3807 proteins were weighted in the HER2+/TN, HR+/TN and HER2+/HR+ networks. Edge weights, $w\left( {\text{e}} \right)$, for all $e \in E$ were then defined as follows:

$$w\left( e \right) = \left\{ {\begin{array}{*{20}c} { \overline{m} \quad \textrm{if} \quad \overline{m} \le 1 } \\ { \frac{1}{{\overline{m}}} \quad \textrm{if} \quad \overline{m} > 1} \\ \end{array} } \right.$$

and where $\overline{m}$ is the mean fold-change of connected vertices, *v*_*1*_ and *v*_*2*,_ (i.e., the mean of *f*(*v*_1_) and *f*(*v*_2_)).

### Measuring the clustering of protein annotations in a weighted PPI network

The objective of PIGNON is to identify annotations for which the associated proteins show a level of differential expression and are clustered in a PPI network. In this study we focus on functional annotations derived from Gene Ontology [10]. For every GO term *T* out of the set of GO terms *O,* we measure the clustering of its associated set of proteins $S_{T}$ and assess their significance. Propagated GO terms, (i.e. where proteins are annotated by all of the parent GO terms of its annotations) were obtained from Himmelstein et al. (accessed 2018-10-26) [56]. PIGNON assesses the clustering of GO terms for which a minimum of 50% of their associated proteins are present in the PPI network and that annotates between 3 and 1000 proteins. This ensures a significant representation of the GO terms analyzed and also that very general GO terms, which typically have little biological interest, are not considered. The clustering of GO terms satisfying these criteria is measured using the total pairwise distance (TPD), a measured inspired by our previously published tool GoNet [29]. Formally, the TPD of the set of proteins $S_{T}$ associated with a GO term *T* is defined as:

$$TPD\left( {S_{T} } \right) = \mathop \sum \limits_{{v_{i} , v_{j} \in S_{T} , i < j}} s\left( {v_{i} ,v_{j} } \right)$$

where $s\left( {v_{i} ,v_{j} } \right)$ is the weighted shortest path distance between the two proteins $v_{i}$ and $v_{j}$. Hence proteins that show an important level of differential expression will be connected with edges with a small weight, thereby reducing their shortest path distances. The weighted shortest path between all pairs of proteins in the network are computed using the Floyd-Warshall algorithm [57, 58].

### Assessing the clustering significance of annotations in an expression-weighted PPI network

To assess the significance of protein clustering of a GO term in a network, PIGNON builds a null model of the TPD for each size of set of proteins $S_{T}$ ranging from 3 to 1000, as described above. Here, we present two methods to build these null models, which are both based on a Monte Carlo sampling strategy. The first is inspired by the sampling strategy from GoNet and randomly samples proteins with a uniform probability, with the exception that only proteins annotated by at least 1 GO term can be sampled [29] and computes the TPD of each sample to build a null distribution of the TPD for a given number of proteins. We will refer to this approach as the unweighted sampling. The second is inspired by another clustering approach, LESMoN [53], and samples proteins with a probability that is roughly proportional to their number of annotations. We will refer to this approach as the weighted sampling. A weighted approach enables the distribution to be more reflective of the impact of proteins that are annotated with multiple GO annotations can have on the clustering results, since such proteins would influence the TPD of multiple GO terms. A null model more often selecting such proteins is therefore likely to be a more accurate model for statistical assessment. For both method 10^7^ random samplings are performed.

The main limitation of these sampling strategies is that they are unable to capture very rare events (i.e. very small TPDs with extremely small *p* values: < 10^–7^). In order to address this issue, one can approximate the null distribution of TPDs using a distribution that has a defined cumulative distribution function. PIGNON therefore computes the TPD mean and standard deviation from the Monte Carlo samples and approximate a normal distribution as the null model. From this normal distribution, we assess the *p* value of obtaining a given TPD α as the probability of obtaining a TPD at least as small as α when randomly selecting a set of proteins in the network.

### Estimating a false discovery rate for a given level of statistical significance

Given the large number of GO terms for which clustering will be assessed, multiple hypothesis testing is an important consideration. However, a large number of GO terms share many annotating proteins and therefore the clustering statistical assessment of these GO terms is far from being independent. Consequently, *p *value correction methods such as Bonferroni are likely too stringent. Furthermore, the approximation using a normal distribution may introduce inaccuracies in the estimation of the statistical significance of a given GO term. Therefore, we restrict reported clustered annotations using an estimated FDR.

In order to estimate this FDR, associations between GO terms and their proteins are shuffled. This shuffling process takes place by selecting at random a pair of GO term-protein association and swapping the protein annotations. This process is repeated 687,855,000 times (a 1000 times the number of GO term-protein associations) in the BioGRID network and 766,371,000 times in the STRING network. For this shuffling, GO terms are not swapped if one of them annotates both proteins in the pair. When this process is completed the clustering statistical significance of the set of shuffled GO terms O* is assessed using the same method as above. We estimate the FDR at a given *p *value threshold *p*, as the proportion of sets of proteins *S*_T*,_ from shuffled GO terms T*, over the number of sets of proteins from actual GO terms, *S*_*T*_*,* with a clustering *p* value smaller than *p.* Specifically,

$$FDR\left( p \right) = \frac{{\mathop \sum \nolimits_{T* \in O*} 1_{{p - value(S_{T*} ){ } < { }p}} }}{{\mathop \sum \nolimits_{T \in O} 1_{{p - value(S_{T} ){ } < { }p}} }}.$$

GO terms, *S*_*T*_*,* for which the proteins are associated with a FDR < 0.001, or the smallest FDR estimated above 0 if this FDR is greater than 0.001, are deemed significantly clustered in an expression-weighted network. Since shuffled GO terms have no biological relevance, their identification provides an estimation of the false discovery rate. This procedure therefore enables a control on the false discoveries that could be generated by PIGNON.

### Benchmarking approaches

#### Unweighted network

PIGNON can be modified such that it solely evaluates the clustering of GO terms and does not consider any differential expression. The network effectively becomes unweighted, where all edges have a weight of 1. The TPD and its statistical assessment can be computed as described above on this unweighted network. PIGNON’s unweighted network analysis is used to contrast PIGNON’s weighted network analysis.

#### Standard functional enrichment analysis

We analyzed the Tyanova et al. dataset using a standard statistical approach, where differentially expressed proteins between the different breast cancer subtypes are detected using a two-tail Student’s *t* test and adjusted for multiple hypothesis testing using the Benjamini–Hochberg method [59]. Ontologizer [14] is then used to statistically assess the level of enrichment of GO terms within the set of significantly differentially expressed proteins, using the entire set of quantified proteins as background. Ontologizer uses a modified Fisher exact test to assess such statistical significance. Two sets of criteria of differential expression were defined: the first required proteins to have a FDR-adjusted *p* value < 0.05, while the second required proteins to have a FDR-adjusted *p *value ≤ 0.20 and a fold change ≤ 0.$\overline{6}$ or ≥ 1.5.

#### Gene set enrichment analysis

The GSEA [13] software was executed to identify GO terms that are differentially regulated between the HER2+/TN, HER2+/HR+ and HR+/TN conditions. GSEA was run with 1000 permutations using the signal to noise of classes as a ranking metric. GO terms of size 3 to 1000 were considered in the analysis. The remaining parameters were set to default values.

#### Network enrichment analysis

We analysed the Tyanova et al. dataset using NEA [31], which identifies enriched functional annotations in gene sets based on a list of significantly differentially expressed proteins using a biological network. We executed this procedure using the NEArender R package (version 1.5) [30] on the BioGRID PPI network and using GO terms as functional annotations. The same criteria for statistically assessing protein differential expression as for the standard functional enrichment analysis were employed. Namely, differentially expressed proteins were defined by an FDR-adjusted *p *value ≤ 0.20 and a fold-change ≤ 0.$\overline{6}$ or ≥ 1.5.

#### Markov clustering algorithm coupled to ontologizer

We compared our PIGNON algorithm to a topological clustering algorithm followed by a GO enrichment analysis. We used the Markov Clustering (MCL) algorithm [36] to identify clusters in the PPI network. MCL was executed on the same BioGRID network produced with the filtering procedures described above with one exception. Edge weights were inverted since MCL clusters proteins based on similarity, not distance. The inflation hyperparameter was set to 2.0. The MCL analysis yielded 1462 clusters containing 3 or more proteins in the HER2+/TN weighted network and 1499 clusters in both the HR+/TN and HER2+/HR+ weighted network. (Additional file 28: File S9). These clusters were analyzed using the GO enrichment analysis tool Ontologizer. Ontologizer assessed the GO term enrichments among the MCL clusters using as background the 16,563 proteins in the BioGRID PPI network.

When correcting for multiple hypothesis testing, we considered each GO term identified in the Ontologizer analysis in all MCL clusters as an individual statistical tests. We performed a Benjamini–Hochberg correction on the resulting *p *values and report the smallest FDR-adjusted *p* value of each GO term.

### Visualization of significant GO term enrichments

We summarized the significant GO terms obtained by each analysis using REVIGO [60] allowing 0.7 SimRel semantic similarity to reduce the redundancy in the reported results. REVIGO’s output is then fed to the CirGO software package [61] to generate a Pie Chart visualization of the results.

### Software availability

PIGNON is implemented as an open-source platform-independent Java program and is available at this address: https://github.com/LavalleeAdamLab/PIGNON. An executable.jar file is also provided along with a “Read Me” documentation.

## Supplementary Information

**Additional file 1: Figure S1**. Approximated normal distributions provide a good estimate of Monte Carlo sampling distributions

**Additional file 2: Figure S2**. PIGNONs FDR performance decreases as significance scores increase

**Additional file 3: Table S1**. PIGNON results on HER2+/TN expression-weighted BioGRID network with weighted sampling

**Additional file 4: Table S2**. PIGNON results on HER2+/TN expression-weighted BioGRID network with unweighted sampling

**Additional file 5: Table S3**. PIGNON results on unweighted BioGRID network with unweighted sampling

**Additional file 6: Table S4**. PIGNON results on unweighted BioGRID network with weighted sampling

**Additional file 7: Table S5**. PIGNON results on HR+/TN expression-weighted BioGRID network with weighted sampling

**Additional file 8: Table S6**. PIGNON results on HER2+/HR+ expression-weighted BioGRID network with weighted sampling

**Additional file 9: Table S7**. PIGNON results on HER2+/TN expression-weighted STRING network with weighted sampling

**Additional file 10: Table S8**. PIGNON results on HER2+/HR+ expression-weighted STRING network with weighted sampling

**Additional file 11: Table S9**. PIGNON results on HR+/TN expression-weighted STRING network with weighted sampling

**Additional File 12: Table S10**. PIGNON results on unweighted STRING network with weighted sampling

**Additional File 13: Figure S3**. Significantly dysregulated GO terms identified by PIGNON in the HER2+/TN breast cancer subtype comparisons that were unique to the expression-weighted STRING network

**Additional File 14: Figure S4**. Significantly dysregulated GO terms identified by PIGNON in the HER2+/HR+ breast cancer subtype comparisons that were unique to the expression-weighted STRING network

**Additional File 15: Figure S5**. Significantly dysregulated GO terms identified by PIGNON in the HR2+/TN breast cancer subtype comparisons that were unique to the expression-weighted STRING network

**Additional File 16: File S1**. t-test results on protein expression values from Tyanova et al.’s dataset

**Additional File 17: File S2**. Ontologizer results of significantly dysregulated proteins from t-test results in Tyanova et al.’s dataset

**Additional File 18: File S3**. Ontologizer results of significantly dysregulated proteins with loosened-up cut-offs from t-test results in Tyanova et al.’s dataset

**Additional File 19: File S4**. GSEA results on Tyanova et al.’s dataset

**Additional File 20: File S5**. NEA results on Tyanova et al.’s dataset using the BioGRID network

**Additional File 21: File S6**. Ontologizer results on clusters identified by MCL with >= 3 proteins in the HER2+/TN expression-weighted BioGRID network

**Additional File 22: File S7**. Ontologizer results on clusters identified by MCL with >= 3 proteins in the HR+/TN expression-weighted BioGRID network

**Additional File 23: File S8**. Ontologizer results on clusters identified by MCL with >= 3 proteins in the HER2+/HR+ expression-weighted BioGRID network

**Additional File 24: Figure S6**. Biological processes identified by the MCL-Ontologizer approach and uniquely detected by PIGNON in the expression-weighted BioGRID networks for all comparisons of breast cancer subtypes

**Additional File 25: Figure S7**. Cellular components identified by the MCL-Ontologizer approach and uniquely detected by PIGNON in the expression-weighted BioGRID networks for all comparisons of breast cancer subtypes

**Additional File 26: Figure S8**. Cellular components identified by PIGNON in breast cancer subtype comparisons that were unique to the expression-weighted BioGRID networks

**Additional File 27: Figure S9**. Molecular functions identified by PIGNON in breast cancer subtype comparisons that were unique to the expression-weighted BioGRID networks

**Additional File 28: File S9**. Protein clusters identified by Markov Clustering Algorithm with >=3 proteins in breast cancer subtype expression-weighted BioGRID networks

## Data Availability

All data generated during this study are included in this published article and its supplementary information files. The datasets analysed during the current study are available in the BioGRID PPI repository, https://downloads.thebiogrid.org/BioGRID/Release-Archive/BIOGRID-3.4.161/ and the STRING PPI repository, https://string-db.org/cgi/download?sessionId=%24input-%3E%7BsessionId%7D&species_text=Homo+sapiens. The quantitative proteomics data (Tyanova et al. [35]) that support the findings of this study are available from Nature Communications, https://www.nature.com/articles/ncomms10259.

## Abbreviations

GO: Gene ontology
PPI: Protein–protein interaction
HR+: Hormone receptor positive
HER2+: Human estrogen receptor 2 positive
TN: Triple negative breast cancer
TPD: Total pairwise distance
FDR: False discovery rate
MCL: Markov Clustering
NEA: Network Enrichment Analysis
GSEA: Gene Set Enrichment Analysis
