# Supplementary material for: PIGNON: a protein–protein interaction-guided functional enrichment analysis for quantitative proteomics
Source: BMC Bioinformatics. 2021 Jun 4;22:302. doi: 10.1186/s12859-021-04042-6 (PMC8178832; doi:10.1186/s12859-021-04042-6)
Supplement: Supplementary file 1 — Additional file 1: Figure S1. Approximated normal distributions provide a good estimate of Monte Carlo sampling distributions [file 12859_2021_4042_MOESM1_ESM.pdf]

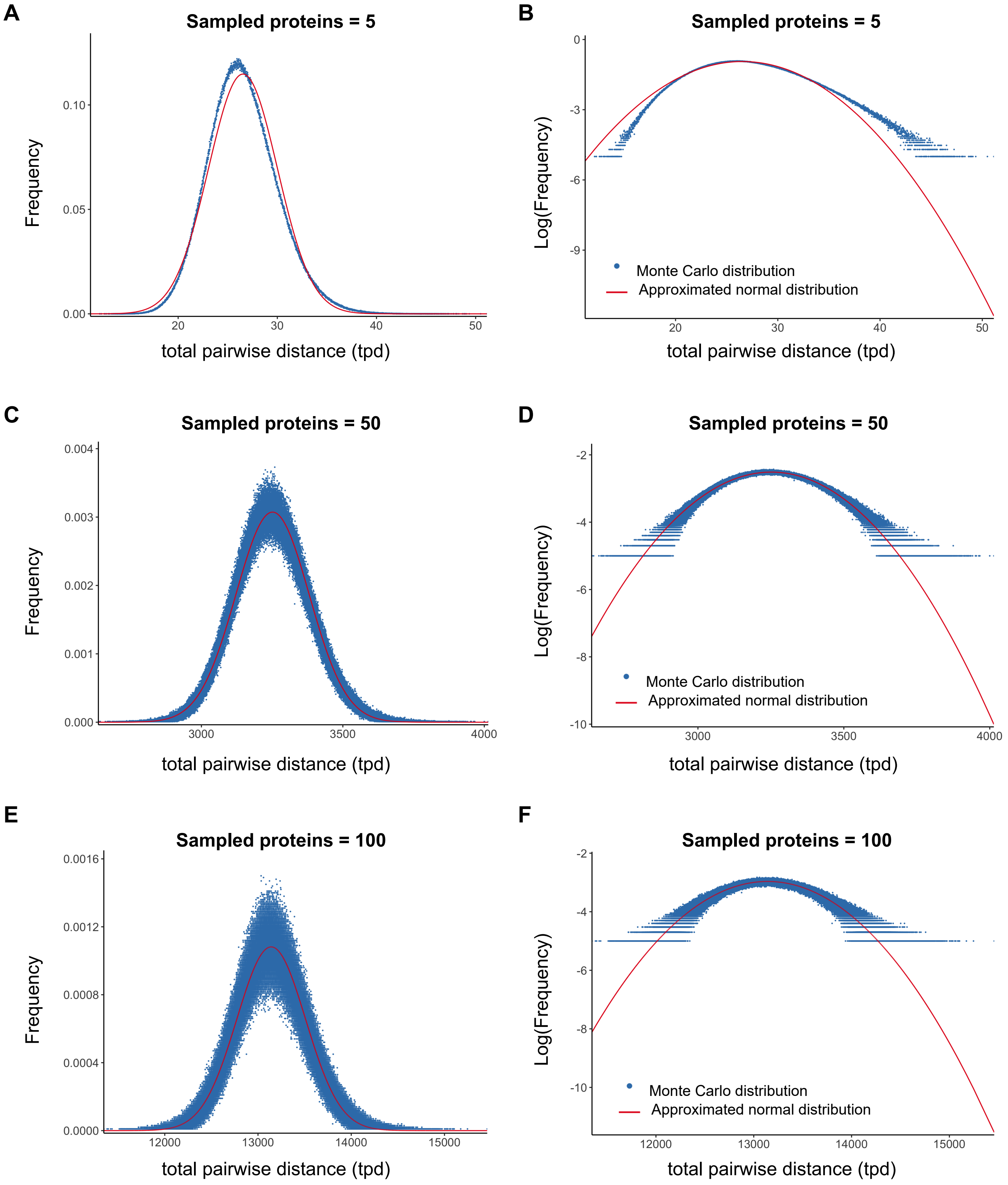

Supplementary Figure S1: Approximated normal distributions provide a good estimate of Monte Carlo sampling distributions. Comparing the TPD frequencies for sample size of n = 5, 50, 100 (A,C,E) and TPD logged frequencies of sample size n = 5, 50, 100 (B,D,F) using Monte Carlo sampling and the approximated normal distributions**.**
