# Supplementary material for: PIGNON: a protein–protein interaction-guided functional enrichment analysis for quantitative proteomics
Source: BMC Bioinformatics. 2021 Jun 4;22:302. doi: 10.1186/s12859-021-04042-6 (PMC8178832; doi:10.1186/s12859-021-04042-6)
Supplement: Supplementary file 2 — Additional file 2: Figure S2. PIGNONs FDR performance decreases as significance scores increase [file 12859_2021_4042_MOESM2_ESM.pdf]

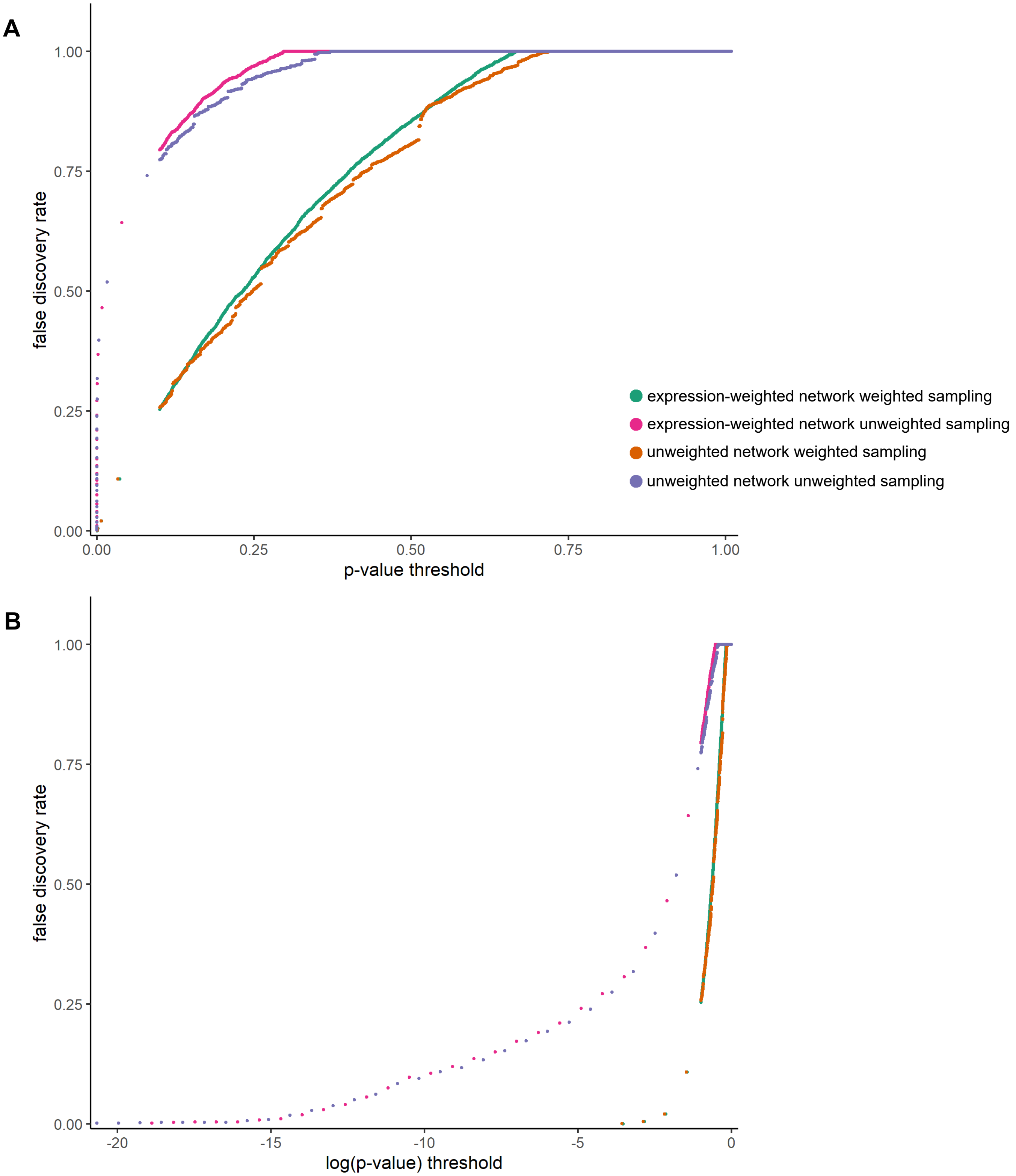

Supplementary Figure S2: PIGNONs FDR performance decreases as significance scores increase. FDRs calculated at various (A) p-value and (B) logged p-value thresholds for the various implementations of PIGNON: HER2+/TN expression-weighted network with unweighted sampling, the HER2+/TN expression-weighted network with weighted sampling, the unweighted network with unweighted sampling, and the unweighted network with weighted sampling.
