# Supplementary material for: PIGNON: a protein–protein interaction-guided functional enrichment analysis for quantitative proteomics
Source: BMC Bioinformatics. 2021 Jun 4;22:302. doi: 10.1186/s12859-021-04042-6 (PMC8178832; doi:10.1186/s12859-021-04042-6)
Supplement: Supplementary file 15 — Additional File 15: Figure S5. Significantly dysregulated GO terms identified by PIGNON in the HR2+/TN breast cancer subtype comparisons that were unique to the expression-weighted STRING network [file 12859_2021_4042_MOESM15_ESM.pdf]

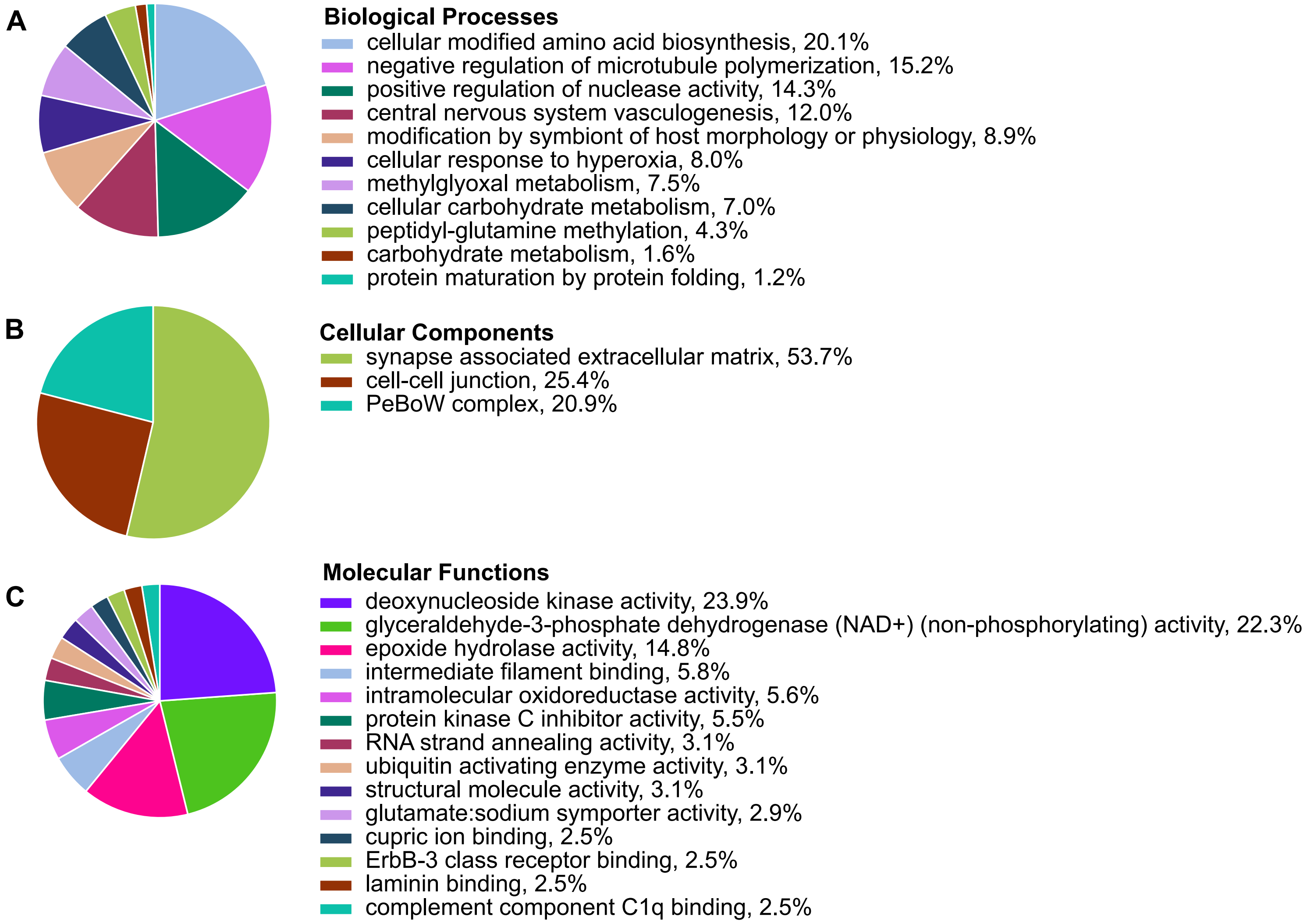

Supplementary Figure S5: Significantly dysregulated GO terms identified by PIGNON in the HR+/TN breast cancer subtype comparisons that were unique to the expression-weighted STRING network. CirGO visualization for (A) biological processes, (B) cellular components and (C) molecular functions that are uniquely identified by PIGNON (FDR < 0.001). The sizes of the pieces of the pies are proportional to the level of enrichment statistical significance and are also denoted as percentages next to the GO term names.
