# Supplementary material for: PIGNON: a protein–protein interaction-guided functional enrichment analysis for quantitative proteomics
Source: BMC Bioinformatics. 2021 Jun 4;22:302. doi: 10.1186/s12859-021-04042-6 (PMC8178832; doi:10.1186/s12859-021-04042-6)
Supplement: Supplementary file 25 — Additional File 25: Figure S7. Cellular components identified by the MCL-Ontologizer approach and uniquely detected by PIGNON in the expression-weighted BioGRID networks for all comparisons of breast cancer subtypes [file 12859_2021_4042_MOESM25_ESM.pdf]

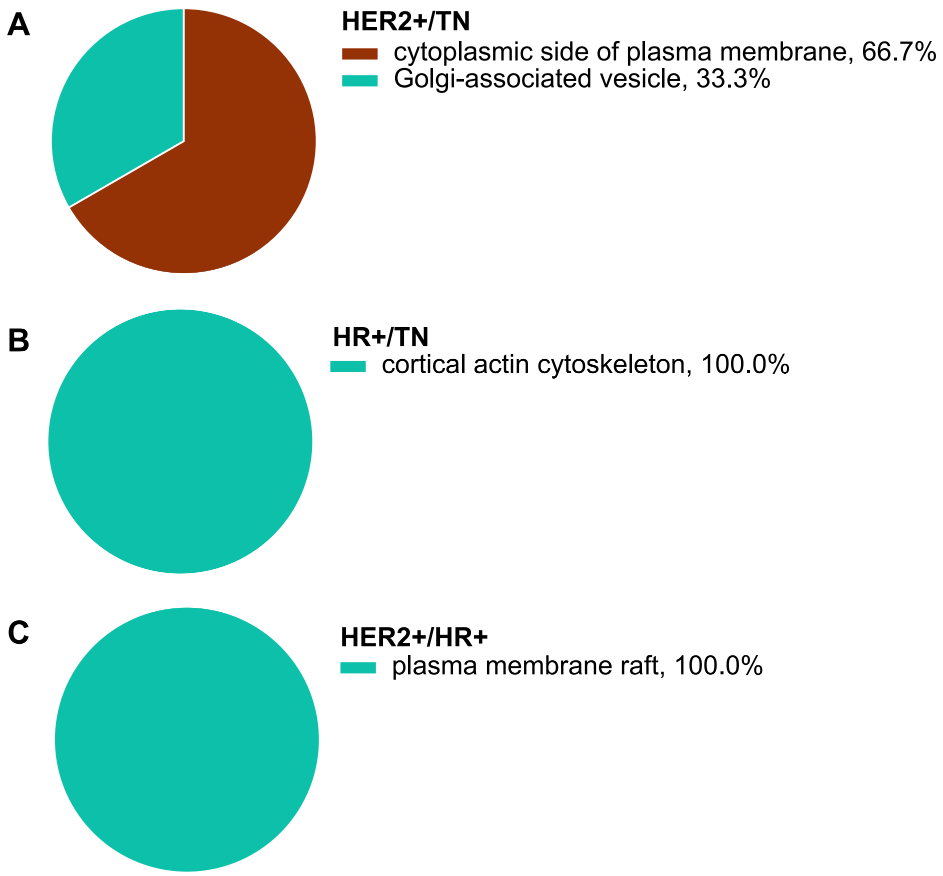

Supplementary Figure S7: Cellular components identified by the MCL-Ontologizer approach and uniquely detected by PIGNON in the expression-weighted BioGRID networks for all comparisons of breast cancer subtypes. CirGO visualization of cellular components in the (A) HER2+/TN (PIGNON FDR < 0.001 and MCL-Ontologizer FDR-adjusted p-value < 0.001), (B) HR+/TN (PIGNON FDR < 0.029 and MCL-Ontologizer FDR-adjusted p-value < 0.029), and (C) HER2+/HR+ (PIGNON FDR < 0.0013 and MCL-Ontologizer FDR-adjusted p-value < 0.0013) expression-weighted networks. The size of the pieces of the pies are proportional to the level of enrichment statistical significance and are also denoted as percentages next to the GO term names.
