# Supplementary material for: PIGNON: a protein–protein interaction-guided functional enrichment analysis for quantitative proteomics
Source: BMC Bioinformatics. 2021 Jun 4;22:302. doi: 10.1186/s12859-021-04042-6 (PMC8178832; doi:10.1186/s12859-021-04042-6)
Supplement: Supplementary file 26 — Additional File 26: Figure S8. Cellular components identified by PIGNON in breast cancer subtype comparisons that were unique to the expression-weighted BioGRID networks [file 12859_2021_4042_MOESM26_ESM.pdf]

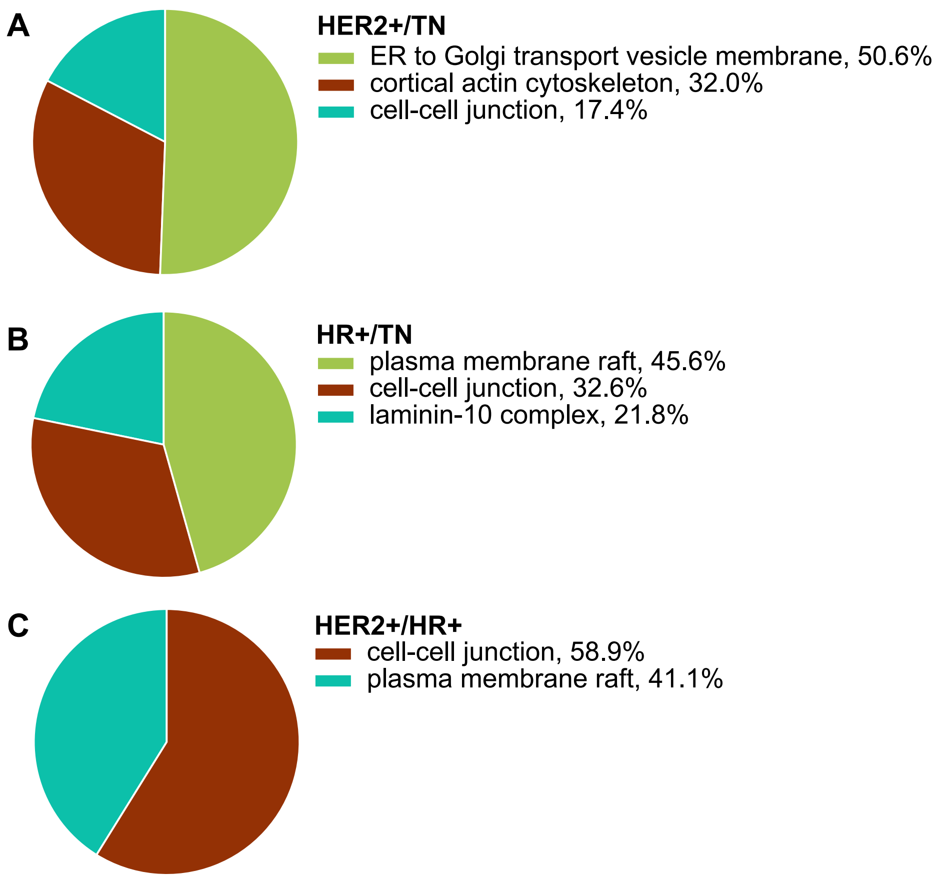

Supplementary Figure S8: Cellular components identified by PIGNON in breast cancer subtype comparisons that were unique to the expression-weighted BioGRID networks. CirGO visualization of uniquely identified cellular components in (A) HER2+/TN (FDR < 0.001), (B) HR+/TN (FDR < 0.0029), and (C) HER2/HR+ (FDR < 0.0013) expression-weighted BioGRID networks. The size of the pieces of the pies are proportional to the level of enrichment statistical significance and are also denoted as percentages next to the GO term names**.**
