# Supplementary material for: PIGNON: a protein–protein interaction-guided functional enrichment analysis for quantitative proteomics
Source: BMC Bioinformatics. 2021 Jun 4;22:302. doi: 10.1186/s12859-021-04042-6 (PMC8178832; doi:10.1186/s12859-021-04042-6)
Supplement: Supplementary file 27 — Additional File 27: Figure S9. Molecular functions identified by PIGNON in breast cancer subtype comparisons that were unique to the expression-weighted BioGRID networks [file 12859_2021_4042_MOESM27_ESM.pdf]

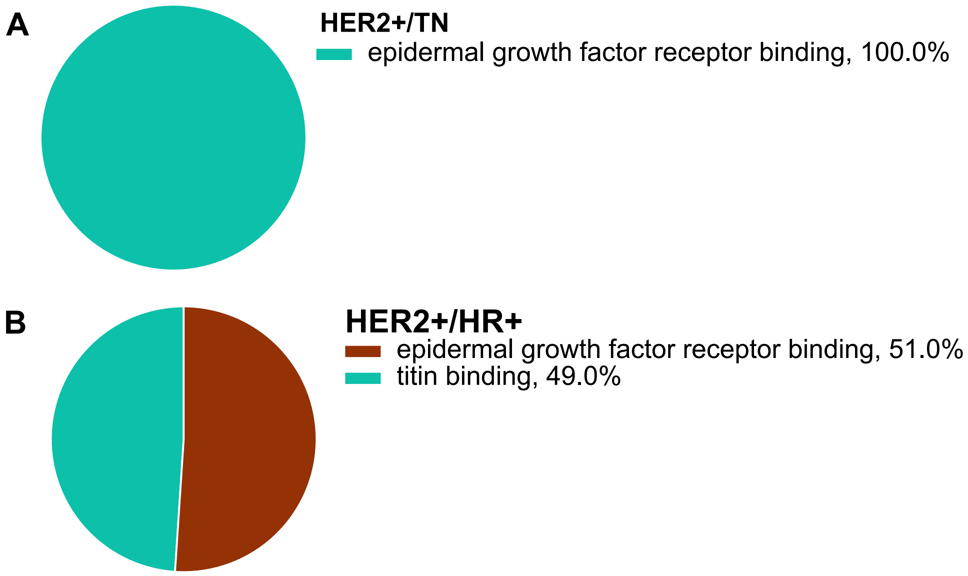

Supplementary Figure S9: Molecular functions identified by PIGNON in breast cancer subtype comparisons that were unique to the expression-weighted BioGRID networks. CirGO visualization of uniquely identified molecular functions in (A) HER2+/TN (FDR < 0.001) and (B) HR+/TN (FDR < 0.0029) expression-weighted BioGRID networks. The size of the pieces of the pies are proportional to the level of enrichment statistical significance and are also denoted as percentages next to the GO term names.
